# Contrast enema in suspected post-NEC intestinal strictures without direct stenosis signs: a retrospective study of 191 surgically confirmed cases

**DOI:** 10.3389/fped.2026.1794956

**Published:** 2026-04-14

**Authors:** Jinfeng Hou, Yong Qin, Qingshuang Liu, Jinping Hou, Yi Wang, Zhenhua Guo, Wei Liu, Shengde Wu

**Affiliations:** 1 Department of General Surgery & Neonatal Surgery, Children’s Hospital of Chongqing Medical University, National Clinical Research Center for Child Health and Disorders, Ministry of Education Key Laboratory of Child Development and Disorders, Chongqing Key Laboratory of Structural Birth Defect and Reconstruction, Chongqing Key Laboratory of Pediatrics, Chongqing, China; 2 Radiology Department, Children’s Hospital of Chongqing Medical University, National Clinical Research Center for Child Health and Disorders, Ministry of Education Key Laboratory of Child Development and Disorders, Chongqing Key Laboratory of Structural Birth Defect and Reconstruction, Chongqing Key Laboratory of Pediatrics, Chongqing, China; 3 Urology Department, Children’s Hospital of Chongqing Medical University, National Clinical Research Center for Child Health and Disorders, Ministry of Education Key Laboratory of Child Development and Disorders, Chongqing Key Laboratory of Structural Birth Defect and Reconstruction, Chongqing Key Laboratory of Pediatrics, Children Urogenital Development and Tissue Engineering of Chongqing Education Commission of China, Chongqing, China

**Keywords:** contrast enema, infant, intestinal stricture, necrotizing enterocolitis (NEC), small bowel obstruction

## Abstract

**Objective:**

Contrast enema (CE) is widely used for suspected post-NEC intestinal strictures, yet some surgically confirmed strictures show no direct stenosis on CE. We aimed to characterize clinical and CE findings in these cases and identify features associated with multi-segment involvement to inform preoperative management.

**Methods:**

We retrospectively reviewed 191 infants with surgically confirmed post-NEC intestinal strictures who underwent preoperative CE. Infants were classified as CE-positive (direct stenosis on CE) or CE-negative (no direct stenosis). Based on intraoperative findings, strictures were further categorized as single-segment or multi-segment. Clinical characteristics and radiographic signs were compared between groups.

**Results:**

Of 191 infants, 153 were CE-positive and 38 were CE-negative. CE-negative infants had a higher rate of prematurity (78.95% vs. 52.94%, *P* = 0.004) and lower birth weight (median 1960 g vs. 2,530 g, *P* = 0.001). CE-negative strictures more frequently involved the ileum and right colon, with a markedly higher rate of isolated small-bowel involvement (39.47% vs. 1.31%, *P* < 0.001). Indirect radiographic signs were common in CE-negative infants, including small-bowel dilatation (73.68% vs. 25.49%, *P* < 0.001) and microcolon (39.47% vs. 1.31%, *P* < 0.001). Bead-like/sausage-like appearance was independently associated with multi-segment involvement (OR = 24.90, *P* < 0.001).

**Conclusion:**

In surgically confirmed post-NEC strictures without direct stenosis on CE, infants are more often premature with lower birth weight, and lesions tend to involve the ileum and right colon. Indirect CE signs such as small-bowel dilatation and microcolon may support clinical decision-making in CE-negative cases, and bead-like/sausage-like appearance suggests a high likelihood of multi-segment involvement.

## 1 Introduction

NEC is one of the most devastating gastrointestinal emergencies in neonates. Up to 47% of survivors develop intestinal strictures after the acute phase, leading to long-term complications such as recurrent vomiting, abdominal distension, feeding intolerance, and malnutrition, severely impacting prognosis and quality of life (1–4). Contrast enema is widely used for evaluating suspected post-NEC strictures or as a routine screening before stoma closure and is one of the primary imaging modalities in most centers (2, 5). However, reported sensitivity and specificity vary across studies, suggesting that contrast enema-negative post-NEC strictures are not uncommon and may lead to delays or misdiagnosis. This highlights the need to identify clinical and radiographic clues to improve diagnostic efficiency. Previous studies have assessed the location and multifocal nature of post-NEC strictures, finding that colonic involvement is common (6), but over 20% of strictures affect the ileum (7), and nearly 20% involve multiple segments (8). These characteristics further complicate preoperative imaging assessment and increase demands for intraoperative exploration and surgical planning. Therefore, identifying potential or multifocal strictures in contrast enema-negative but highly suspicious cases through clinical features and indirect imaging signs (e.g., proximal small bowel dilation, microcolon, bead-like appearance) is of significant clinical relevance. This study retrospectively analyzed the clinical and contrast enema imaging data of 191 infants with post-NEC intestinal strictures, focusing on the clinical features and indirect radiographic signs in contrast enema-negative cases, and describing the typical imaging findings of multi-segment strictures to provide evidence for clinical recognition and diagnosis of post-NEC intestinal strictures.

## 2 Methods

### 2.1 Study design/clinical workflow

**Figure 1:**
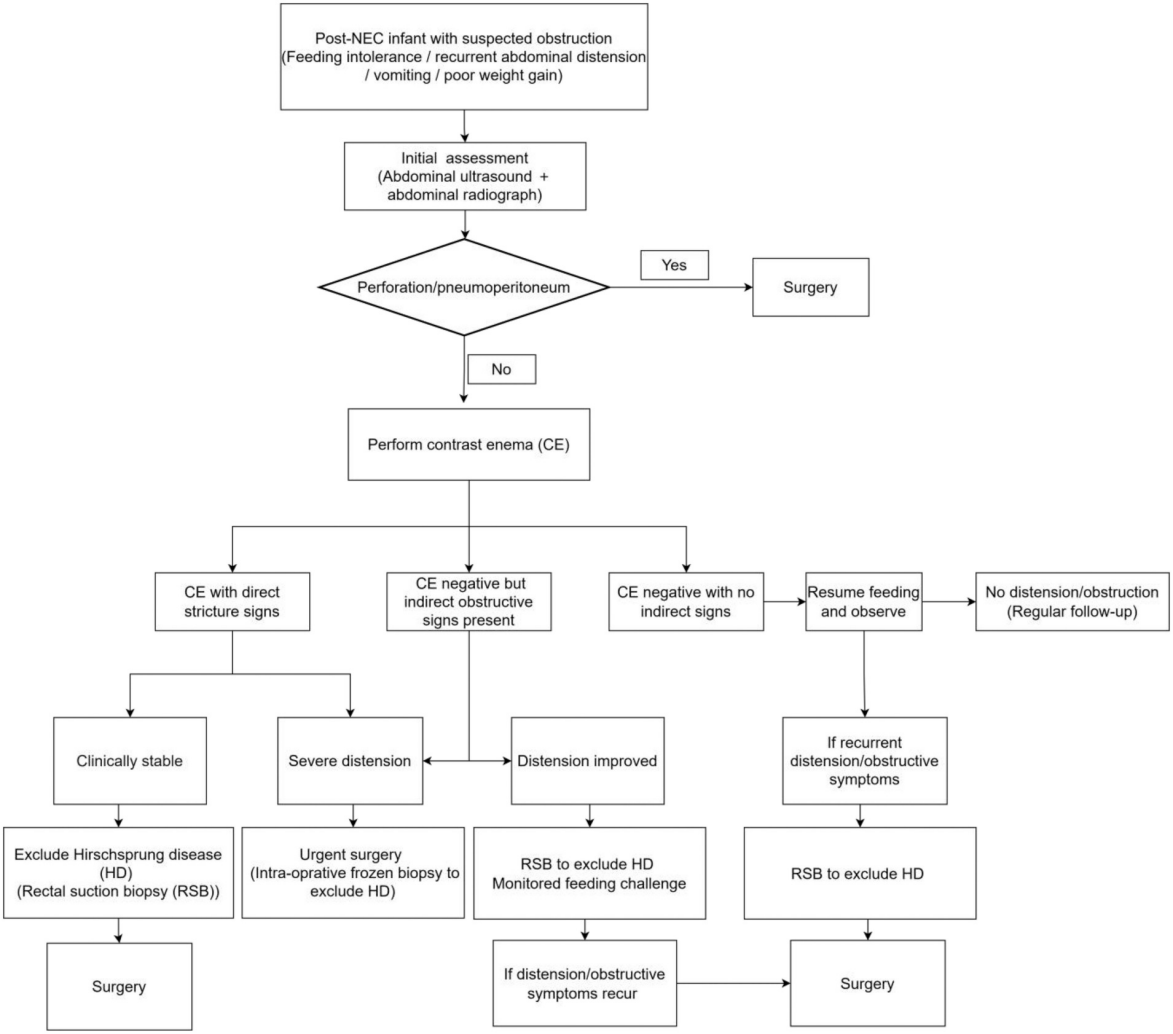
Clinical workflow for evaluation and management of suspected post-NEC intestinal obstruction.

This was a single-center, retrospective cohort study conducted in a pediatric specialty hospital between January 2015 and December 2024. We included infants with surgically confirmed post-NEC intestinal strictures who underwent preoperative contrast enema after initial conservative management. The clinical workflow used for preoperative evaluation and decision-making is summarized in Figure 1.

### 2.2 Inclusion and exclusion criteria

#### 2.2.1 Inclusion criteria

(1) Infants diagnosed with NEC who developed bowel obstruction or feeding intolerance symptoms (e.g., vomiting, abdominal distension, cessation of bowel movements) and had surgical confirmation of intestinal stricture after conservative treatment. (2) Preoperative contrast enema performed. (3) Complete clinical and imaging data.

#### 2.2.2 Exclusion criteria

(1) Patients who underwent surgery during the acute phase of NEC. (2) Patients with severe congenital bowel malformations, such as congenital megacolon. (3) Contrast enema studies with unavailable, incomplete, or non-reviewable original imaging data were excluded. In our cohort, this accounted for only a small number of cases (a total of six), mainly because the original images could not be retrieved from the medical record/imaging archive system.

### 2.3 Grouping

Patients were divided into two groups based on contrast enema results:

CE-positive group: Contrast enema showed direct evidence of stricture (linear sign, cutoff sign, conical sign).
CE-negative group: No direct radiographic signs of stricture were identified on contrast enema, whereas post-NEC intestinal stricture was confirmed surgically. In this study, intestinal stricture includes small-bowel stricture, colonic stricture, or combined involvement.

Patients were also categorized into single-segment and multi-segment stricture groups based on intraoperative findings (Multi-segment stricture was defined as two or more distinct strictures in different intestinal segments identified intraoperatively).

### 2.4 Data collection

Patient data were extracted from electronic medical records and imaging archives. Collected variables were categorized as follows: (A) demographics/perinatal factors; (B) clinical presentation; (C) comorbidities; (D) perioperative course and outcomes; (E) laboratory indices (pre- and postoperative); (F) imaging variables (ultrasound and contrast enema signs); and (G) operative/distribution variables (stricture location and segment involvement). The complete list of collected variables is provided in Tables 1–3.

**Table 1:** Comparison of clinical and laboratory characteristics between patients with and without contrast enema–detected intestinal stricture.

Variable	Contrast enema-positive (*n* = 153)	Contrast enema-negative (*n* = 38)	*χ*²/t/Z	*P* value
Sex (male: female)	89:64	16:22	3.174^a^	0.075
Blood type (A, B, O, AB)	A: 58; B: 38; O: 48; AB: 9	A: 15; B: 11; O: 10; AB: 2	0.500^a^	0.919
Preoperative mechanical ventilation	18 (11.76%)	14 (36.84%)	13.725^a^	**<0** **.** **001**
Bronchopulmonary dysplasia	3 (1.96%)	3 (7.89%)	1.842^a^	0.175
Preoperative blood transfusion	86 (56.21%)	29 (76.32%)	5.137^a^	**0**.**023**
Mode of delivery (vaginal vs. cesarean)	Vaginal: 46 (30.07%) Cesarean: 107 (69.93%)	Vaginal: 8 (21.05%) Cesarean: 30 (78.95%)	1.219^a^	0.270
Twin birth	45 (29.41%)	11 (28.95%)	0.003^a^	0.955
*In vitro* fertilization	11 (7.19%)	2 (5.26%)	0.004^a^	0.950
Premature birth	81 (52.94%)	30 (78.95%)	8.458^a^	**0**.**004**
History of hypoxia	16 (10.46%)	6 (15.79%)	0.407^a^	0.524
Feeding history (breast, formula, mixed)	Breast: 37 (24.18%) Formula: 70 (45.75%) Mixed: 46 (30.07%)	Breast: 11 (28.95%) Formula: 20 (52.63%) Mixed: 7 (18.42%)	2.068^a^	0.356
Diarrhea	8 (5.23%)	1 (2.63%)	0.062^a^	0.804
Hematochezia	84 (54.90%)	12 (31.58%)	6.623^a^	**0**.**010**
Abdominal distension	128 (83.67%)	31 (81.58%)	0.095^a^	0.758
Fever	59 (38.56%)	10 (26.32%)	1.987^a^	0.160
Vomiting	66 (43.14%)	18 (47.37%)	0.221^a^	0.638
Feeding intolerance	42 (27.45%)	11 (28.95%)	0.034^a^	0.854
Intraoperative blood transfusion	132 (86.27%)	33 (86.84%)	0.008^a^	0.927
Elevated preoperative CRP	43 (28.10%)	9 (23.68%)	0.300^a^	0.584
Preoperative hypoproteinemia	55 (35.95%)	16 (42.11%)	0.494^a^	0.482
Elevated postoperative CRP	45 (29.41%)	11 (28.95%)	0.003^a^	0.955
Postoperative hypoproteinemia	78 (50.98%)	24 (63.16%)	1.814^a^	0.178
Postoperative blood transfusion	77 (50.33%)	20 (52.63%)	0.065^a^	0.799
Ileal stricture	24 (15.69%)	25 (65.79%)	40.064^a^	**<0**.**001**
Ileocecal–ascending colon involvement	75 (49.02%)	21 (55.26%)	0.475^a^	0.491
Ileum–ascending colon involvement	79 (51.63%)	36 (94.74%)	23.605^a^	**<0**.**001**
Transverse colon involvement	51 (33.33%)	2 (5.26%)	11.963^a^	**0**.**001**
Descending colon involvement	81 (52.94%)	0 (0.00%)	34.932^a^	**<0**.**001**
Sigmoid colon stricture	36 (23.53%)	1 (2.63%)	8.511^a^	**0**.**004**
Multiple segment involvement	75 (49.02%)	12 (31.58%)	3.733^a^	0.053
Small intestine only involved	2 (1.31%)	15 (39.47%)	50.080^a^	**<0**.**001**
Stoma formation	42 (27.45%)	17 (44.74%)	4.261^a^	**0**.**039**
Unplanned reoperation	17 (11.11%)	2 (5.26%)	0.601^a^	0.438
Contrast enema: small intestine dilatation	39 (25.49%)	28 (73.68%)	31.045^a^	**<0**.**001**
Ultrasound suggested stricture*	6/143 (4.20%)	1/35 (2.86%)	Fisher	1.000
Contrast enema: microcolon	2 (1.31%)	15 (39.47%)	50.080^a^	**<0**.**001**
Contrast enema: cecum non-filling	89 (58.17%)	16 (42.11%)	3.174^a^	0.075
Age at presentation (days)	40.00 (16.50, 67.50)	38.50 (8.00, 53.25)	1.156^b^	0.248
Rectal suction biopsy	129 (84.31%)	30 (78.95%)	0.629^a^	0.428
Age at examination (days)	47.00 (34.00, 74.50)	52.00 (40.75, 62.00)	−0.312^b^	0.755
Age at surgery (days)	54.00 (42.00, 78.50)	61.00 (46.50, 69.50)	−0.553^b^	0.581
Interval from examination to surgery (days)	7.00 (3.00, 8.00)	8.00 (3.00, 12.00)	−1.655^b^	0.098
Preoperative hospital stay (days)	13.00 (7.00, 25.00)	20.00 (10.00, 36.50)	−2.211^b^	**0**.**027**
Total hospital stay (days)	30.00 (21.50, 42.50)	35.50 (26.75, 59.00)	−3.098^b^	**0**.**002**
Weight at admission (g)	3,130.00 (2,495.00, 3,800.00)	2,425.00 (1,962.50, 3,102.50)	3.822^b^	**<0**.**001**
Weight at surgery (g)	3,200.00 (2,700.00, 3,865.00)	2,915.00 (2,475.00, 3,157.50)	2.365^b^	**0**.**018**
Birth weight (g)	2,530.00 (2,075.00, 3,100.00)	1,960.00 (1,620.00, 2,602.50)	3.292^b^	**0**.**001**
Gestational age (weeks)	36.48 ± 2.85	33.89 ± 3.44	−4.81^c^	**<0**.**001**
Time to postoperative bowel function recovery (days)	6.00 (5.00, 8.00)	7.00 (5.75, 7.25)	−0.742^b^	0.458
Time to full enteral nutrition (days)	13.00 (12.00, 16.00)	12.50 (11.00, 16.25)	0.528^b^	0.597
Preoperative WBC (×10⁹/L)	10.98 (8.42, 13.61)	8.87 (7.29, 12.56)	2.049^b^	0.04
Preoperative RBC (×10¹²/L)	3.27 ± 0.62	3.33 ± 0.64	0.562^c^	0.575
Preoperative hemoglobin (g/L)	93.00 (84.00, 108.00)	93.50 (86.00, 109.25)	−0.494^b^	0.622
Preoperative platelet count (×10⁹/L)	424.00 (329.00, 592.00)	388.50 (268.50, 513.75)	1.556^b^	0.120
Preoperative albumin (g/L)	32.04 ± 5.30	31.71 ± 5.60	−0.332^c^	0.74
Postoperative WBC (×10⁹/L)	7.83 (5.91, 11.29)	5.43 (4.47, 6.68)	5.098^b^	<0.001
Postoperative RBC (×10¹²/L)	3.53 ± 0.55	3.60 ± 0.59	0.694^c^	0.489
Postoperative hemoglobin (g/L)	102.45 ± 15.97	106.61 ± 18.08	1.397^c^	0.164
Postoperative platelet count (×10⁹/L)	334.65 ± 136.08	263.21 ± 98.17	−3.043^c^	0.003
Postoperative albumin (g/L)	30.15 ± 5.57	28.13 ± 6.12	−1.962^c^	0.051

Bold values indicate selected variables with statistically significant differences between groups that were considered clinically meaningful.

Because some infants had multiple strictures, the total number of stricture sites exceeded the total number of cases.

^a^ Is the χ²-value.

^b^ Is the *Z*-value.

^c^ Is the *t*-value.

* Preoperative ultrasound examination was completed in 178 patients, *p* value was from Fisher's exact test.

**Table 2:** Comparison of cardiac malformations between patients with and without contrast enema–detected intestinal stricture.

Variable	Contrast enema-positive (*n* = 140)	Contrast enema-negative (*n* = 34)	χ²	*P* value
Atrial septal defect	71 (50.71%)	22 (64.71%)	2.152	0.142
Patent ductus arteriosus	8 (5.71%)	0 (0.00%)	0.942	0.332
Ventricular septal defect	19 (13.57%)	8 (23.53%)	2.069	0.150

Only 174 patients underwent echocardiography during hospitalization.

**Table 3:** Comparison of radiological features between patients with single-segment and multi-segment intestinal strictures.

Variable	Multi-segment stricture (*n* = 87)	Single-segment stricture (*n* = 104)	χ²	*P* value
Linear sign	60 (68.97%)	47 (45.19%)	10.86	**0** **.** **001**
Cutoff sign	7 (8.05%)	13 (12.50%)	1.002	0.317
Conical Sign	8 (9.20%)	18 (17.31%)	2.651	0.103
Beaded/Sausage-like sign	37 (42.53%)	3 (2.88%)	44.968	**<0**.**001**
Small bowel dilatation	28 (32.18%)	39 (37.50%)	0.588	0.443
Microcolon	7 (8.05%)	10 (9.62%)	0.144	0.704
Unfilled cecum sign	44 (50.57%)	61 (58.65%)	1.249	0.264

Bold values indicate selected variables with statistically significant differences between groups that were considered clinically meaningful.

**Figure 2:**
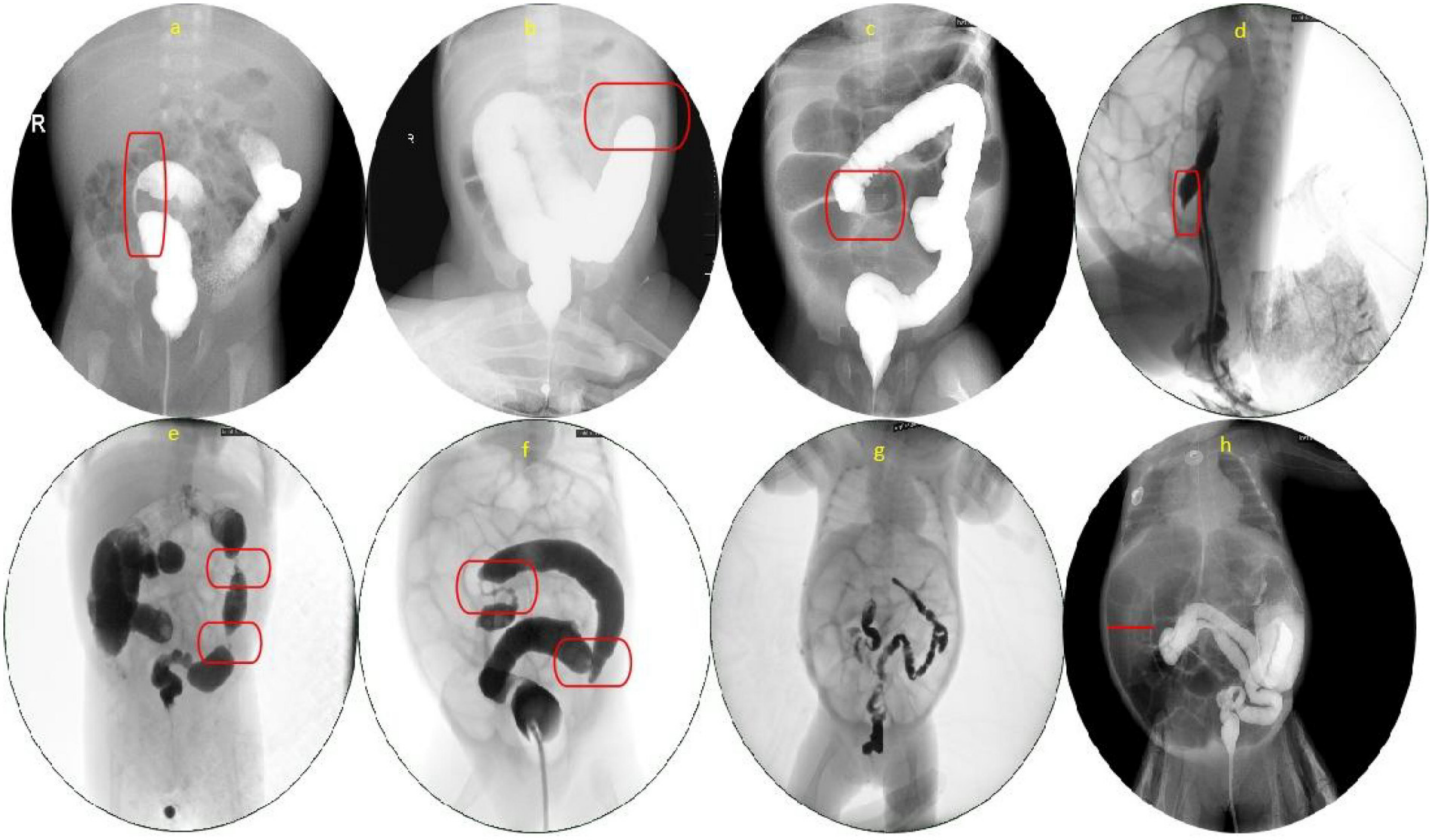
Contrast enema findings of intestinal strictures following NEC. **(a)** Linear Sign: Thread-like passage of contrast through a markedly narrowed segment. **(b)** Cutoff sign: Abrupt termination of the contrast column at the stenotic site. **(c)** Conical sign / unfilled cecum sign: Gradual tapering of the lumen with a non-opacified cecum. **(d)** Conical sign: Proximal narrowing of the contrast column forming a conical tip. **(e)** Bead-like sign: Alternating multiple areas of constriction and dilatation resembling beads. **(f)** Sausage-like sign: The colonic segment between two areas of stenosis appears uniformly narrowed and resembles a sausage when filled with contrast. **(g)** Microcolon: Hypoplastic or underdeveloped colon with reduced caliber. **(h)** Small bowel dilatation: The small intestine is markedly dilated, with a diameter exceeding that of the colon.

Contrast enema (CE) imaging signs evaluated included the linear, cutoff, and conical signs, as well as small-bowel dilatation, microcolon, the unfilled cecum sign, and bead-like/sausage-like appearance (Figure 2). Images were independently assessed by a radiologist and a pediatric surgeon (both associate chief physician or higher); disagreements were resolved by a third senior physician.

### 2.5 Definitions

In this study, “intestinal stricture” refers to post-NEC bowel stricture involving the small intestine, colon, or both.

Contrast enema: Retrograde contrast imaging of the colon and distal small intestine was performed using a diluted barium sulfate suspension (approximately 25%–33% weight per volume) or a water-soluble contrast agent (e.g., iopromide) diluted 1:1 with normal saline, administered via the rectum under fluoroscopic guidance.

Linear Sign: A segment of the bowel is extremely narrowed, outlined by a thin, elongated column of contrast, appearing as a “fine line.”

Cutoff Sign: Contrast within the bowel suddenly terminates at a fixed level, forming a “cutoff” or “abrupt termination” appearance.

Conical Sign: Contrast column gradually tapers toward the stricture/proximal bowel, forming a cone with the apex pointing proximally.

Small Bowel Dilation: The diameter of the proximal small bowel exceeds 2.0–2.5 cm (9–11) or is greater than that of the colon.

Microcolon: The colon appears small in caliber, typically <1 cm in diameter (12), lacking normal colonic haustra or peristalsis (13).

Bead-like/Sausage-like Appearance: Multiple narrowings separated by normal or dilated bowel segments, forming a beaded or sausage-like shape.

Unfilled cecum sign: During contrast enema, the contrast fails to reach or adequately opacify the cecum (14).

**Figure 3:**
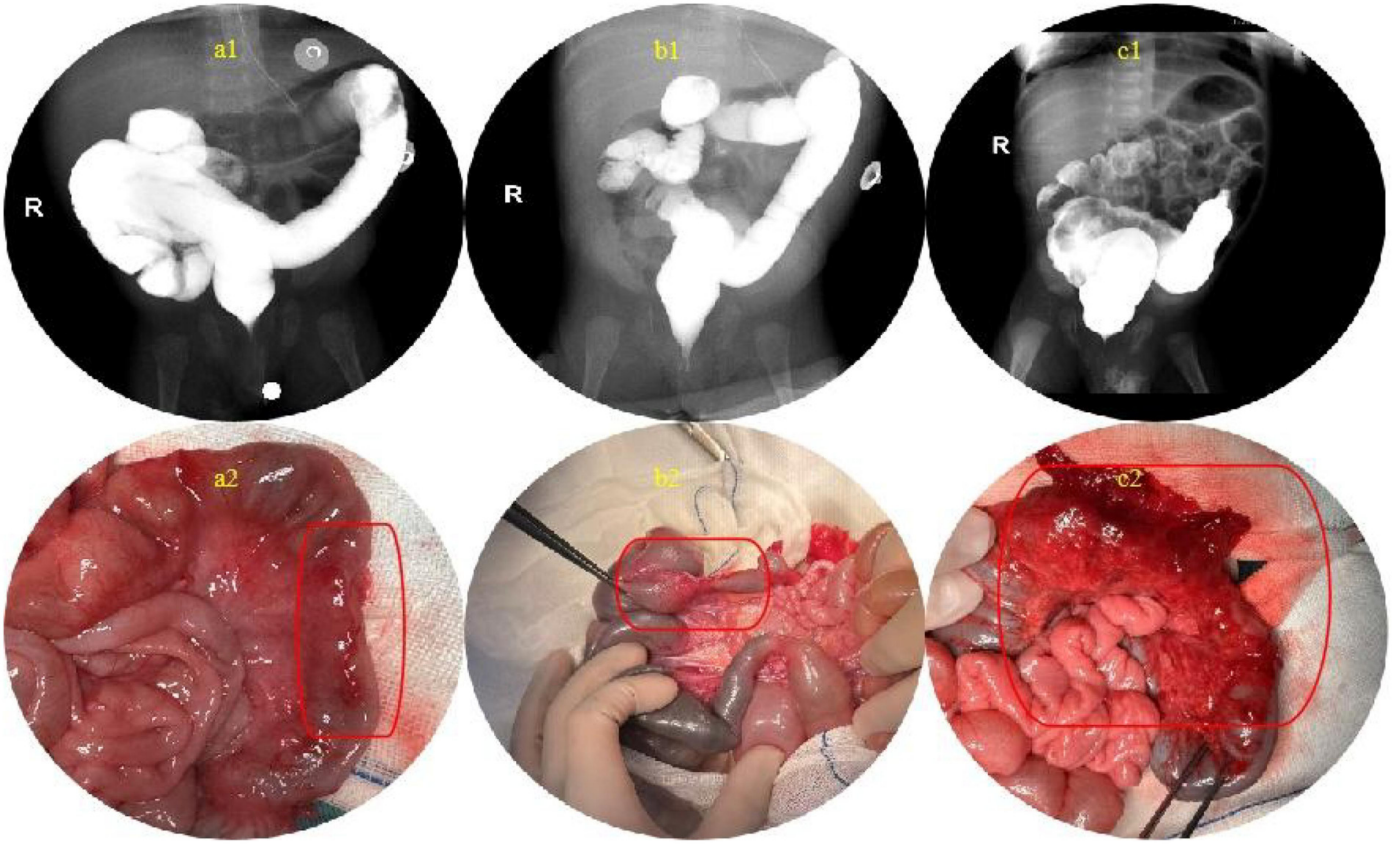
Comparison between contrast enema–negative and contrast enema–positive intestinal stenosis cases. **(a1,b1)** Contrast enema images show normal bowel contour without evident stenosis. **(a2,b2)** Intraoperative findings confirm terminal ileal stenosis. **(c1)** Contrast enema demonstrates a segmental narrowing from the splenic flexure to the descending colon. **(c2)** Surgery confirms stenosis at the same location as shown on contrast study.

These radiographic signs are demonstrated in Figure 2. Figure 3 shows the comparison between contrast enema–negative and contrast enema–positive intestinal stenosis cases.

### 2.6 Statistical analysis

Statistical analysis was performed using SPSS 26.0 software. Continuous data were expressed as mean ± standard deviation (SD) or median (interquartile range, IQR), and categorical data were presented as frequency and percentage. Group comparisons were performed using independent sample *t*-tests or Mann–Whitney *U* tests for continuous variables, and chi-square or Fisher's exact tests for categorical variables. Multivariate logistic regression was used to analyze independent risk factors for multi-segment involvement. A two-sided *P* value of <0.05 was considered statistically significant.

## 3 Results

A total of 191 infants with post-NEC intestinal strictures were included in the study, consisting of 105 males and 86 females. Among them, 111 were preterm and 80 were term infants. The median gestational age was 36.29 (34.14, 38.43) weeks, and the average birth weight was 2,497.27 ± 781.80 g. The median surgical weight was 3,080.00 (2,600.00, 3,700.00) g, and the median hospital stay was 31.00 (23.00, 45.00) days. Of the 191 cases, 153 were positive on contrast enema, and 38 were negative. Rectal suction biopsy was performed in 159/191 (83.25%) infants to exclude Hirschsprung disease, including 30/38 (78.95%) in the CE-negative group and 129/153 (84.31%) in the CE-positive group. No infant was diagnosed with Hirschsprung disease. Strictures involved the ileum in 49 cases, the ileocecal junction to ascending colon in 96 cases, the transverse colon in 53 cases, the descending colon in 81 cases, and the sigmoid colon in 37 cases. Among these, 87 cases had multi-segment involvement, and 17 had isolated small bowel involvement. A total of 59 infants underwent stoma surgery, and 132 infants underwent primary intestinal resection and anastomosis. The overall preoperative evaluation and management pathway used in our cohort is illustrated in Figure 1.

### 3.1 Clinical and imaging results of CE-negative cases

Compared to the positive group, the negative group (*N* = 38) had a higher rate of prematurity (78.95% vs. 52.94%, *χ*² = 8.458, *P* = 0.004), smaller gestational age (33.89 ± 3.44 weeks vs. 36.48 ± 2.85 weeks, t = −4.81, *P* < 0.001), and lower birth weight [median 1,960 (1,620, 2,602.5) g vs. 2,530 (2,075, 3,100) g, Z = 3.292, *P* = 0.001]. Admission weight and surgical weight were also lower in the negative group (admission weight 2,425 (1,962.5, 3,102.5) g vs. 3,130 (2,495, 3,800) g, Z = 3.822, *P* < 0.001; surgical weight 2,915 (2,475, 3,157.5) g vs. 3,200 (2,700, 3,865) g, Z = 2.365, *P* = 0.018). In terms of perioperative factors, the negative group had a higher rate of preoperative ventilator use (36.84% vs. 11.76%, *χ*² = 13.725, *P* < 0.001) and preoperative blood transfusions (76.32% vs. 56.21%, *χ*² = 5.137, *P* = 0.023), longer preoperative hospitalization [20 (10, 36.5) days vs. 13 (7, 25) days, Z = −2.211, *P* = 0.027], and longer total hospital stay [35.5 (26.75, 59) days vs. 30 (21.5, 42.5) days, Z = −3.098, *P* = 0.002]. Blood stool was more common in the positive group (54.90% vs. 31.58%, *χ*² = 6.623, *P* = 0.010), while other symptoms such as abdominal distension, vomiting, and fever showed no significant differences. Imaging findings revealed that the negative group was more likely to have small bowel or right colon involvement: ileal involvement was significantly higher (65.8% vs. 15.7%, *χ*² = 40.064, *P* < 0.001), and ileocecal to ascending colon involvement was more common (94.7% vs. 51.6%, *χ*² = 23.605, *P* < 0.001). Left colon involvement (transverse, descending, sigmoid) was significantly lower in the negative group (transverse colon 5.3% vs. 33.3%, *P* = 0.001; descending colon 0% vs. 52.9%, *P* < 0.001; sigmoid colon 2.6% vs. 23.5%, *P* = 0.004). Isolated small bowel strictures were more common in the negative group (38.5% vs. 1.3%, *χ*² = 54.686, *P* < 0.001). The negative group also had a higher rate of stoma formation (44.7% vs. 27.5%, *χ*² = 4.261, *P* = 0.039), while the rate of unplanned reoperation was not significantly different (*P* = 0.438). Regarding contrast enema imaging, the negative group was more likely to show small bowel dilation (73.7% vs. 25.5%, *χ*² = 31.045, *P* < 0.001) and microcolon (39.5% vs. 1.3%, *χ*² = 50.080, *P* < 0.001) (Table 1). As shown in Table 2, no difference in cardiac malformation incidence was found between the two groups.

### 3.2 Imaging results of multi-segment stricture cases

Intraoperative findings stratified by segment involvement showed that multi-segment strictures (*N* = 87) were more likely to present with linear signs (69.0% vs. 45.2%, *χ*² = 10.86, *P* = 0.001) and bead-like/sausage-like signs (42.5% vs. 2.9%, *χ*² = 44.968, *P* < 0.001) (Table 3).

Using multi-segment involvement (0 = No, 1 = Yes) as the dependent variable, and seven imaging features as independent variables, stepwise binary logistic regression was performed. The results showed that only the bead-like/sausage-like appearance entered the final model (B = 3.215, SE = 0.625, Wald = 26.492, *P* < 0.001). The odds ratio (OR) was 24.9 (95% CI: 7.3–84.8), indicating that the presence of bead-like/sausage-like appearance significantly increases the risk of multi-segment involvement, making it an independent risk factor (Table 4). The logistic regression model using bead-like/sausage-like appearance to predict multi-segment involvement was as follows: logit(p) = −0.703 + 3.215 × (Bead-like/Sausage-like Appearance), imaging features were coded as binary variables (0 = absent, 1 = present). The area under the ROC curve (AUC) was 0.698 (95% CI: 0.621–0.776, *P* < 0.001), indicating moderate discriminatory ability of the model, which is reasonable considering it was based on a single imaging feature.

**Table 4:** Logistic regression analysis of radiological features associated with multi-segment intestinal stricture (forward stepwise method).

Variable	B	SE	Wald χ²	*P* value	OR	95% CI
Beaded/Sausage-like sign	3.215	0.625	26.492	<0.001	24.9	7.3–84.8
Constant	−0.703	0.173	16.531	<0.001	0.495	0.353–0.695

## 4 Discussion

In our cohort, contrast enema-negative post-NEC strictures occurred predominantly in more premature infants with lower birth and surgical weights and were associated with greater preoperative support needs. These findings suggest that these infants have poorer baseline physiological reserves and more complex conditions. The negative group also showed a higher incidence of ileal to right colon involvement, consistent with literature reporting that contrast enema sensitivity for diagnosing small bowel strictures is 0.667 (15), leading to more frequent misdiagnosis of small bowel strictures (16). A negative contrast enema result does not necessarily exclude post-NEC intestinal stricture, because contrast enema primarily evaluates the colon and the contrast medium may not pass through the ileocecal valve into the ileum in some infants. In such cases, ileal or other small-bowel strictures may not be directly visualized, resulting in false-negative findings. In cases of insufficient cecal filling during contrast enema, right colon and ileocecal junction strictures can also be missed (17). These findings provide both population- and anatomical-based explanations for why some cases are negative, with contrast enema showing higher sensitivity for colonic stricture after NEC but insufficient sensitivity for small bowel involvement. A study on contrast enema after NEC ostomy closure found that contrast enema accurately diagnosed distal bowel strictures with 100% sensitivity and 97% specificity (18). Another study of post-NEC intestinal strictures reported a contrast enema sensitivity of 93.9% and specificity of 93.8% (19), though the risk of both false negatives and positives remains. Because our cohort consisted of surgically confirmed strictures and did not include non-stricture controls after contrast enema, the present study focuses on the distribution and indirect radiographic patterns in CE-negative cases rather than estimating diagnostic accuracy.

In addition, the 59 infants who underwent stoma creation in our cohort were not a separate group in whom stricture was incidentally identified only at the time of stoma closure. They were also patients with confirmed post-NEC intestinal stricture requiring surgical treatment. Although bowel resection with primary anastomosis is generally the preferred approach, stoma creation may be selected as a staged operative strategy in high-risk situations, such as a short interval between NEC and stricture diagnosis with concern for persistent inflammation, low body weight with increased risk of anastomotic leakage, inability to completely exclude Hirschsprung disease, marked discrepancy between proximal and distal bowel caliber, or emergency presentation with intestinal obstruction or sepsis. Therefore, the higher rate of stoma formation in CE-negative cases likely reflects greater clinical complexity and operative risk rather than a different disease category.

Hematochezia is a common clinical sign in post-NEC intestinal stricture patients (1). This study found that the incidence of hematochezia was significantly higher in the contrast enema-positive group compared to the negative group, likely due to differences in bowel function and the physical state of the intestinal contents. In the contrast enema-positive group, more colonic strictures were observed, while the negative group mainly had small bowel strictures. In the small intestine, liquid contents are less likely to cause mechanical damage to the stricture wall and dilute any minor bleeding, making visible hematochezia less likely. In contrast, in the colon, solid or semi-solid feces can cause more mechanical damage to the stricture wall, resulting in overt bleeding.

Small bowel strictures account for approximately one-third of all post-NEC intestinal strictures (7, 20). Contrast enema's sensitivity for small bowel strictures is relatively low, as direct signs such as linear, cutoff, and conical signs are less common and often missed. In this study, the negative cases exhibited significant enrichment of small bowel dilation and “microcolon” signs, suggesting that indirect signs can indicate potential obstruction and distal dysfunction even in the absence of direct stricture signs. The characteristic “microcolon” reflects a state of chronic underfilling or disuse of the colon, with potential causes including total colonic aganglionosis or small bowel atresia (12). When ileal strictures are almost occlusive, microcolon may appear. Small bowel dilation also suggests distal obstruction (21). In post-NEC infants, if contrast enema shows microcolon or small bowel dilation along with symptoms like abdominal distension and vomiting, NEC-related strictures should be highly suspected, and early surgical exploration should be considered to prevent severe complications like recurrent infections, sepsis, or bowel perforation (17). Notably, 9 cases in this study had no direct or indirect stricture signs on contrast enema (Figure 2). After excluding congenital megacolon (negative rectal biopsy), surgery confirmed strictures, with 8 cases involving the ileum to ascending colon and 1 involving the sigmoid colon. The latter was misdiagnosed as congenital megacolon because the stricture location was still visible on contrast enema, and the proximal colon was notably dilated. These results highlight that, despite its importance, contrast enema has diagnostic limitations, especially in small bowel and right colon strictures, and involves unavoidable ionizing radiation. Therefore, exploring higher diagnostic efficacy imaging strategies and combined assessment methods, such as ultrasound or MRI, is warranted (22–25).

From an imaging perspective, the linear sign reflects a highly narrowed bowel lumen forming a “fine line” of contrast (26, 27), which is the most common direct sign of post-NEC intestinal stricture, accounting for 69.93% of direct stricture signs. The bead-like/sausage-like appearance suggests “segmented strictures” with normal or dilated bowel segments between narrowings (28), correlating with multi-segment involvement both biologically and radiologically. When a contrast enema image shows linear signs indicative of “severe local narrowing,” and bead-like/sausage-like changes indicate “multiple lesions,” clinical awareness of multi-segment involvement should be heightened. It is important not to focus solely on the most obvious stricture as the single surgical target and to consider other affected segments. While the presence of bead-like/sausage-like signs suggests multi-segment involvement, the absence of these signs does not exclude it, especially when cutoff or conical signs are present, as contrast may not show the shape of distal bowel segments. Therefore, all bowel segments should be carefully examined during surgery, with saline infusion into the distal bowel to assess filling status and detect hidden strictures.

Previous studies on post-NEC colonic strictures have shown that high-frequency ultrasound can diagnose colonic strictures with 98.0% sensitivity and 100% specificity (19), offering a low-radiation, bedside repeatable supplement. Ultrasound was performed in most infants, but bowel gas and abdominal distension frequently limited visualization. In our cohort, ultrasound suggested stricture in only 7/178 infants (3.93%), with no difference between CE-positive and CE-negative groups (Table 1), precluding a robust head-to-head comparison with contrast enema.

## 5 Limitations

This study has several limitations. First, it was a retrospective single-center study, which may limit the generalizability of the findings. Second, infants who underwent contrast enema but were ultimately not diagnosed with intestinal stricture (e.g., those who improved without surgery) were not included as a control group. As a result, conventional diagnostic performance measures for specific imaging features, such as sensitivity, specificity, positive predictive value, and negative predictive value, could not be reliably calculated. In addition, although abdominal ultrasound was frequently performed, bowel gas related to abdominal distension often limited sonographic visualization, resulting in a relatively low ultrasound-positive rate; therefore, a meaningful head-to-head comparison between ultrasound and contrast enema was not feasible. Further multicenter prospective studies are needed to validate these findings.

## 6 Conclusions

In infants with surgically confirmed post-NEC intestinal strictures, CE-negative cases were more often premature with lower birth weight and more frequently involved the ileum/right colon or isolated small bowel. When no direct stenosis is identified on CE, indirect signs such as small-bowel dilatation and microcolon may help support clinical assessment. Bead-like or sausage-like appearance was strongly associated with multi-segment involvement and may help inform operative planning. The proposed workflow (Figure 1) may provide a structured framework for evaluating suspected post-NEC obstruction in infants.

## Data Availability

The data supporting the findings of this study are available from the corresponding author upon reasonable request.
